# Interventions for Type 2 Diabetes Prevention and Management Among Indigenous Children and Youth: A Systematic Review

**DOI:** 10.1002/edm2.70026

**Published:** 2025-01-13

**Authors:** Edmund Wedam Kanmiki, Yaqoot Fatima, Thuy Linh Duong, Roslyn Von Senden, Tolassa W. Ushula, Abdullah A. Mamun

**Affiliations:** ^1^ Poche Centre for Indigenous Health The University of Queensland Brisbane Queensland Australia; ^2^ ARC Centre of Excellence for Children and Families Over the Life Course (Life Course Centre) The University of Queensland Brisbane Queensland Australia; ^3^ Faculty of Nursing and Midwifery Hanoi Medical University Hanoi Vietnam; ^4^ School of Exercise and Nutrition Sciences, Faculty of Health Deakin University Melbourne Australia

**Keywords:** Culturally appropriate interventions, Early prevention, Indigenous populations, Type 2 diabetes, Youth

## Abstract

**Introduction:**

Indigenous populations experience a disproportionately higher burden of early onset of type 2 diabetes mellitus (T2DM). To contribute towards addressing this health disparity, evidence‐based culturally appropriate interventions are urgently needed. This systematic review examines interventions designed to improve the prevention and management of T2DM among Indigenous children and youth.

**Methods:**

A comprehensive search of five electronic databases was carried out in February 2023 to identify relevant studies published in English. We included studies of all designs involving Indigenous children and youth under 25 years of age. An adapted version of the National Institute of Health (NIH) quality assessment tool for pre‐post intervention studies was used for quality assessment. Due to the heterogeneity of methods used by reviewed publications, the convergent integrated approach developed by Joanna Briggs Institute (JBI) for mixed‐method systematic reviews was employed in the analysis. Prospero registration ID: CRD42023423671.

**Results:**

The search identified 1127 publications, and 25 studies with a total of 4594 participants from four countries were eligible after screening. Notably, most (80%) originated from North America. Most interventions involved < 100 participants and lasted 6 months or less (58%). While knowledge and behaviours improved for most interventions, longer and culturally responsive interventions, often combining both community and school‐based elements, demonstrated a greater effect on key anthropometrics and biomarkers associated with the risk of T2DM.

**Conclusion:**

This review highlights the urgent need for more research to address T2DM among Indigenous youth. Future research should prioritise culturally appropriate, long‐term interventions that engage communities and empower Indigenous youth to make healthy choices.

## Graphical abstract

This systematic review examines interventions used in preventing and managing type 2 diabetes among Indigenous children and youth. Most interventions were short‐term and focused on improving knowledge and behaviour change. Longer, culturally appropriate interventions, combining community and school‐based approaches, showed greater promise in reducing diabetes risk factors. Future research should prioritise long‐term, culturally appropriate interventions that empower Indigenous youth to make healthy choices.

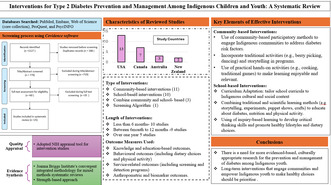

## 1 Introduction

Indigenous peoples are culturally distinct people, societies and communities who share ancestral ties to the lands, waters and natural resources where they live, or from which they have been displaced [1]. Due to systemic inequities, the historical effect of colonisation and inadequate culturally appropriate public health interventions, Indigenous people face higher health inequalities including higher rates of health risks, poorer health outcomes and greater unmet needs in terms of health and social services [2].

Although the global burden of type 2 diabetes mellitus (T2DM) is well‐recognised in middle‐aged and older adults [3], Indigenous peoples including Indigenous children and young adults experience higher rates of early onset of T2DM [4, 5, 6, 7, 8]. For instance, T2DM is more prevalent both in young and older Indigenous Australians compared to non‐Indigenous Australians [5, 9]. Similarly, there is a higher prevalence of T2DM among Indigenous Māori and Indigenous Indian and Alaska Natives compared to the general population in New Zealand and the USA, respectively [4, 7].

Early onset of T2DM leads to longer exposure to higher blood glucose levels and increases diabetes‐related complications and comorbidities. Such long‐term exposure and related adverse effects can substantially affect young people, their families and the community as well as strain resources within the health system [9]. These effects also reduce the quality of life and productivity of affected people during their prime ages and potentially shorten their life expectancy. In addition, due to the association of T2DM with insulin resistance, it also elevates the risk of other cardiovascular diseases, including hypertension, high cholesterol, stroke and myocardial infarction [10].

Culturally appropriate programs that build on the strengths and knowledge of Indigenous communities are needed to prevent and manage T2DM among Indigenous populations. This may include strategies for promoting healthy lifestyles such as optimising diet, physical activity, avoiding smoking and enhancing access to quality healthcare including screening and early disease detection.

Inadequate evidence‐based culturally responsive interventions hinder T2DM prevention and management in Indigenous youth. This review examines existing interventions for preventing and managing T2DM among Indigenous youth (< 25 years) to: investigate and categorise the existing T2DM management and preventive strategies, examine the effectiveness of those strategies and identify the characteristics of effective interventions.

## 2 Materials and Methods

### 2.1 Search Strategy and Selection Criteria

A systematic literature search was conducted on 18 February 2023, across five databases (PubMed, Embase, Web of Science, ProQuest and PsycINFO) to identify research on T2DM prevention or management in Indigenous youth under the age of 25 years. This age group was selected because it represents a critical developmental period where both brain development and the potential adoption of unhealthy behaviours such as poor dietary choices, sedentary lifestyles and substance use, can easily be developed and have lifelong health consequences.

Keywords and synonyms related to Indigenous people, youth, diabetes (including type 2 diabetes), prevention, management, intervention and programs were used. Boolean operators and appropriate Mesh terms were incorporated to refine the search. Table S1 provides a complete list of search terms used. Google Scholar and reference list of relevant publications were checked for possible publications meeting the inclusion criterion. This study followed the Preferred Reporting Items for Systematic Reviews and Meta‐Analyses (PRISMA) framework [11, 12]. PRISMA is a comprehensive framework that guides the transparent conduct and reporting of systematic reviews and meta‐analysis. Furthermore, a strength‐based approach aligning with Indigenous peoples' worldviews has guided the conduct of this review [13].

### 2.2 Inclusion and Exclusion Criteria

Studies were included if they focused on interventions aimed at preventing or managing T2DM among Indigenous youth under 25 years, regardless of study design or publication date. Studies were excluded if they did not target Indigenous youth, T2DM prevention/management or lack of effectiveness evaluation. Review articles and studies that focus solely on risk factors were also excluded. Studies not published in English language were also excluded.

## 3 Data Analysis

**FIGURE 1:**
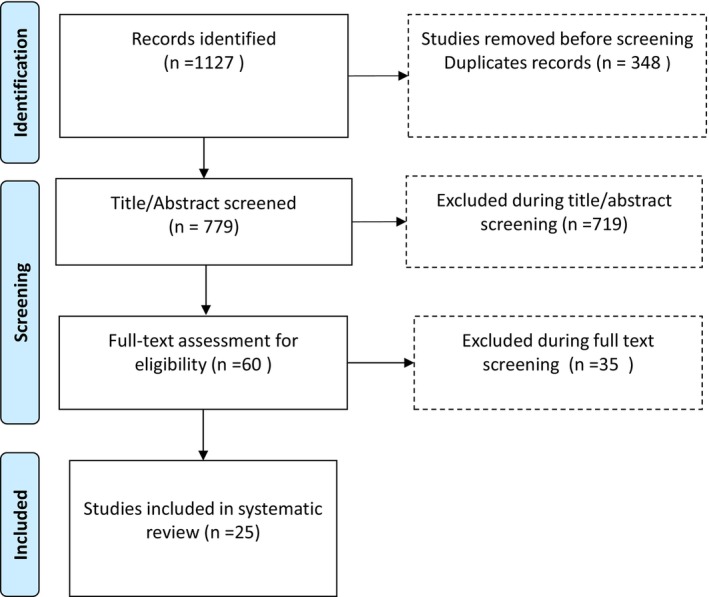
PRISMA flow chart.

All citations retrieved from databases were imported into *Covidence software* for screening. Duplicates were removed automatically. Titles and abstracts were screened for relevance. Full texts of articles were then reviewed for final inclusion based on the pre‐defined criteria. Figure 1 presents the PRISMA flowchart showing the screening process. Screening was done by two independent reviewers (E.W.K. and T.W.U.). The final publications included in the analysis are in Table 1. Extracted data includes publication year, country, study objective, design, characteristics of participants, brief description of intervention, methods used and outcomes measures.

**TABLE 1:** General characteristics of the studies included in the systematic review.

Study	Country	Objective	Study design	Participants: sex; age in years (mean or range)	Intervention	Follow‐up period and retention	Data analysis method and confounders	Outcome measures and results related to T2DM
Aho et al. 2011 [14]	USA	Testing a culturally appropriate curriculum for T2D preventive education and healthy lifestyles among American Indian youth	Longitudinal study Mix methods	1500 students of both sexes from kindergarten through 12th grade; elementary (*n* = 386), middle (*n* = 893) and high school (*n* = 240)	School‐based: K–12 Diabetes Education in Tribal Schools (DETS) is a state‐of‐the‐art science curriculum developed by the Diabetes Prevention Program (DPP) considering the cultural context of American Indians	Approx. 6‐months (retention information not clear)	Quant data paired *t*‐test. Did not include cofounders Qual data: narrative accounts	Outcome measures: Knowledge on T2DM, healthy diet choices, physical activity, traditional knowledge and values around food choices. Results: intervention increased knowledge about T2DM and healthy diet choices and activities in all grade levels (Elementary, Middle and High school).
Brown et al. 2013 [15]	USA	Develop a lifestyle change program and assess implementation indicators concerning short‐term behavioural and physiological outcomes	Longitudinal study Quantitative	64 Native American youth: sexes 10–14 years old. 32 were randomised to the ‘Journey DPP’ group, while the remaining 32 were randomised to the comparison group	Community‐based: Modifications of DPP (Journey DPP) included incorporating cultural aspects and delivering the program in small groups led by community members	3‐months 84% retention rate	Paired *t*‐tests and no adjustments made (crude change only)	Outcome measures: BMI, dietary intake, physical activity and nutrition knowledge, attitudes and beliefs. Results: Improved measures of PA, nutrition knowledge, attitude and behaviour score in Journey DPP. The intervention did not affect overall BMI, but those with overweight/obesity at baseline may be more sensitive to the intervention
Carrel et al. 2005 [16]	USA	Improve lifestyles (diet and PA) of children at high risk for T2DM.	Longitudinal study	38 obese (BMI > 85th percentile) Ho‐Chunk and non‐native children (proportion and gender not reported); age 6–18 years	Combination of school and community‐based intervention: The Ho‐Chunk Youth Fitness Project. Twice per week, supervised nutrition and exercise classes were divided into fitness and nutrition education, PA (45 min) and making a healthy snack (30 min)	6‐months 100% retention rate	Mean test No control for confounders	Outcome measures: Insulin, glucose, cholesterol and body fat Results: Improved fasting insulin after the intervention. The intervention did not affect body fat percentage, blood glucose or total cholesterol levels
Chadwick et al. 2019 [17]	USA	Increase PA and estimate its impact on clinical measures related to health and fitness through collaborative work between community tribal leaders and academic staff (Feasibility study)	Randomised treatment	77 American Indian youth with overweight/obesity: both sexes; age 11–20 years	Community‐based: Incentive Theory of Motivation principles. Youth were incentivised to complete exercise sessions during three randomised treatment phases. Each phase had two incentive groups: a control and an increasing incentive group	16 weeks for each phase 100% retention rate	Not stated	Outcome measures: Feasibility of intervention Results: Through early, frequent and open conversations between tribal leaders and academic investigators, culturally appropriate and tribal directed randomised clinical trial to increase PA in American Indian youth was effectively implemented within a rural tribal area. Whether this intervention had improved clinical outcomes is not stated.
Chambers et al. [18]	USA	Examine the effect of engaging adult caregivers in youth diabetes prevention programs (Feasibility study)	Longitudinal study Quantitative	226 (88% of 256 youths: both sexes; age 10–19 years) enrolled with designated adult caregivers (18 years and above). Only 37 caregivers had anthropometric data collected over time	Community‐based: Home‐visiting program (Together on Diabetes TOD) consisting of 12 lessons for youth and 4 for caregivers and delivered by Native American paraprofessional family health coaches	12‐months 100% retention rate	Paired *t*‐tests (assessed caregiver physiologic changes between baseline (an average of data from baseline and 3 months) and at 12 months) as evaluation time points	Outcome measures: feasibility, acceptability and effectiveness (BMI, waist circumference) Results: Engaging adult caregivers in youth diabetes prevention programs was feasible, acceptable and effective. Also, available data on physical measurements for a subset of adult caregivers show that the program had beneficial effects on the body weight and BMI of these adult caregivers.
Chansavang et al. 2015 [19]	New Zealand	Improve healthy lifestyle to improve cardiorespiratory fitness (VO_2_), health and usual activity in Pacific and Māori children	Longitudinal study Quantitative	18 secondary school student participants	School‐based: After‐school exercise and lifestyle programme for less‐active Pacific and Māori adolescents. Three times weekly 1.5 h exercise and healthy lifestyle sessions	6‐weeks 89% retention rate	Paired *t*‐tests	Outcome measures: cardiorespiratory fitness (VO2max), insulin resistance (Homeostasis Model Assessment), physical activity, glycated haemoglobin (HbA1c), fasting plasma glucose, blood pressure, waist circumference and fasting lipids. Results: Improvements in VO_2_ max (3.2 mL/kg/min); SBP (−10.6 mmHg), HbA1c (−1.1 mmol/mol) and weekly vigorous (4 h) and moderate (2 h) physical activity. However, waist circumference increased during a follow‐up
Colip et al. 2016 [20]	USA	Promote healthy eating and regular exercise to prevent T2DM	Longitudinal study	65 adolescent Zuni Indians: both sexes; mean age, 13.9 ± 1.7 years	Community‐based: Tri‐weekly exercise session (60 min) with a monthly diet and nutritional counselling session. An instructional session for parents on healthy eating and preparing nutritional sack lunches for their children.	24 weeks Retention ranges from 46.2% to 61.5% based on outcomes evaluated	Paired *t*‐tests and intention to treat.	Outcome measures: metabolic profile (fasting lipids, A1c), vital signs (blood pressure, resting heart rate) and anthropometric characteristics Results: Reduced body weight and BMI as well as per cent body fat and increased fat‐free mass. Reduced blood biomarkers, including HbA1c, FBG, TC, LDL, TG and increased HD
Ducharme‐Smith et al. 2021 [21]	USA	Improve behavioural factors (diet quality) and evaluate their association with cardiometabolic risk factors	Longitudinal study	240 youth diagnosed with T2DM or prediabetes were identified as at risk based on BMI and laboratory tests: both sexes, ages 10 and 19 years	Community‐based: Together on Diabetes (TOD) program. TOD is home‐based biweekly nutrition education (12 lessons for 45–60 min during a 6‐month intervention phase followed by 20‐min maintenance lessons every 6 months) delivered to youth and their enrolled adult caregiver	12 months 80% retention rate	Linear mixed‐effects models (adjusted for age, sex, study site, energy intake and food security). Further, for height for blood pressure and diabetes, medication use for HbA1c, with depression and physical activity also included for blood pressure and HbA1c	Outcome measures: AHEI‐2010 score Results: No change in diet quality, but reduced reduction in energy, sugar‐sweetened beverages, fluid, red/processed meat and sodium. Despite stable diet quality scores over time, the improvement in diet quality domains is likely associated with a reduced HbA1c among youth with diabetes. The diet did not affect blood pressure and BMI
Eskicioglu et al. 2014 [22]	Canada	Improve healthy living behaviours, knowledge, self‐efficacy and anthropometric measures	Quasi‐experimental trial with a parallel nonequivalent control arm	151 grade 4 and 5 students: both sexes; mean age 9.7 years (51 received the intervention, and 100 were in the control group)	School‐based: Curriculum: Aboriginal Youth Mentorship Program (AYMP). Once a week, 90 min, after‐school peer‐led program on physical Activity (healthy play), nutrition (healthy food), education and healthy relationships for fourth‐grade students (*n* = 51) facilitated by high school mentors. The control group (students in grade 5, *n* = 100) received the standard curriculum	Five months 100% retention rate	Linear mixed effects models (adjusted for baseline age, gender and adiposity). Multiple linear regression analysis (changes in waist circumference and BMI z‐score)	Outcome measures: knowledge of healthy foods, waist circumference, BMI Results: Improved knowledge of healthy foods and body image in the intervention arm. Children in the intervention arm gained less waist circumference and BMI *z*‐score, and this finding persisted for waist circumference when the analysis was restricted to those with overweight and obesity, indicators of interventions, change in self‐efficacy was found to be the best predictor of change in waist circumference in the multivariable‐adjusted linear regression model
Frejuk et al. 2021 [23]	Canada	Improve laboratory testing in screened patients for chronic diseases	Longitudinal study	324 children: both sexes, age 10–17 years	Community‐based: The FINISHED program is a community‐engaging mobile mass screening program targeting chronic diseases, including T2DM, in people 10years or older. Controls were from the same community using propensity score analysis	18 months 100% retention rate	Difference‐in‐differences models to estimate group differences in change of screening and primary care follow‐up frequency over time. Adjusting for confounders not stated.	Outcome measures: effect on diabetes screening Results: Improved laboratory testing in screened patients for T2DM
Huynh et al. 2015 [24]	Canada	Assess the feasibility and lived experiences of an intensive group‐based lifestyle intervention focusing on improving diet, increasing PA and reducing weight to manage T2DM	Longitudinal study	12 overweight/obese youth with T2DM: both sexes; aged 13–19 years	Community‐based: Beating Diabetes Together: Intensive lifestyle interventions involving two 90‐min sessions per week. An established curriculum (Bright Bodies) that addresses nutrition education, PA and practical activities (e.g., cooking) based on conventional behaviour‐modification techniques, motivational interviewing and peer mentoring	16 weeks 75% retention rate	Paired *t*‐tests	Outcome measures: feasibility and effects on glycaemic control, blood pressure and anthropometric measures Results: The intervention was feasible but did not significantly affect glycaemic control, blood pressure and anthropometric measures
Kakekagumick et al. 2013 [25]	Canada	Promote healthy eating and exercise to prevent the onset of diabetes	Longitudinal study	138 students from kindergarten to grade 5: both sexes and age	Combination of school and community‐based intervention: The Sandy Lake school‐based diabetes curriculum, part of the Sandy Lake Health and Diabetes Project (SLHDP), is the main component of school intervention. Biweekly lessons incorporate taste tests, skill building, goal setting, humour, games, intergenerational learning and storytelling using Aboriginal characters	17 weeks 88.4% retention rate	Not stated	Outcome measures: effect on dietary choices, knowledge, screen time, VO_2_ max and anthropometric measurements Results: Evaluation I: 1998–1999. Increased dietary intention, dietary preference, knowledge of curriculum concepts, dietary self‐efficacy and decreased screen time. Evaluation II: 2005–2006: Improved self‐efficacy, knowledge of health and nutrition, and screen time did not impact VO_2_ max and anthropometric measurements
Macaulay et al. 1997 [26]	Canada	Improve healthy eating and increase PA in elementary school children to prevent early onset diabetes (feasibility study)	Note stated clearly stated, presumably quasi‐experimental trial with a parallel nonequivalent control arm	458 children in grades 1–6 (260 attended the English school, comparison group 198 participated at the Mohawk immersion school, intervention group); both sexes, ages 6–12 years	Combination of school and community‐based intervention: The Kahnawake Schools Diabetes Prevention Project (KSDPP). A health education program for children in grades 1–6 containing sections on nutrition, fitness, diabetes, understanding the human body and healthy lifestyles in 10 lessons (45 min each) per year for each grade	3 years Retention rate not reported (results based on only baseline survey)	Descriptives	Outcome measures: Feasibility Results: The program was feasible through participatory research incorporating Native culture and local expertise based on baseline reports
Malseed et al. 2014 [27]	Australia	Improve knowledge, attitudes, self‐efficacy and behaviours of urban Indigenous young people regarding chronic disease and its risk factors	Longitudinal study (controlled)	79 students, majority (approx. 89% Indigenous): both sexes; aged 11–18 years	School‐based: Deadly Choice, a school‐based health promotion and education program, was delivered weekly at six education facilities in Brisbane, Australia, to participants from grades 7–12 (intervention group: grade 8; *n* = 16) over time	Seven weeks Retention rate not reported and cannot be inferred from the paper	Mixed effect models. All models were adjusted for repeated measures at the individual level, with the time interaction term used. Paired *t‐*test	Outcome measures: knowledge, attitudes, self‐efficacy, PA, breakfast, intake of fruits and vegetables, health checks Results: more improvements in knowledge, attitudes, self‐efficacy and behaviours; breakfast frequency, PA frequency, fruit and vegetable intake for the intervention group than the control groups
Manifold et al. 2019 [28]	Australia	Increasing T2DM screening in adolescents visiting PHC services using a simplified screening algorithm incorporating HbA1c	Pilot study	50 children: both sexes, aged 10–14 years	Screening algorithm: A locally developed screening algorithm that adopted recommendations from a Baker Heart and Diabetes Institute position statement on T2DM screening in young Indigenous Australians, the ADA 2015 and the Kimberley T2DM guideline for adults.	6‐month pilot period 56% retention rate	Fisher's exact test	Outcome measures: effect on the number of T2DM screening Results: More patients received an initial T2DM screening test at the Aboriginal Community Controlled Health Service. Still, there was no change at the hospital during the pilot (27 June–26 December 2016) compared to a baseline (i.e., six months before the pilot), (1 October 2015–31 March 2016)
Marlow et al. 1998 [29]	USA	Developing culturally sensitive education programs to prevent early onset diabetes	Longitudinal study	24 adolescents: both sexes; age 13–18 years	Community‐based: Stop Diabetes! Program; provides information about diabetes and its prevention through physical activity and good nutrition within a cultural context particularly for Native Americans. Four motivated adolescents, self‐named the Coyotes, assisted in the development and leadership of the program	3 months 100% retention rate	Not stated	Outcome measures: knowledge about diabetes, nutrition and exercise Results: Increased knowledge about diabetes, nutrition and exercise. The program was found to be effective, suggesting the importance of culturally appropriate interventions to prevent T2DM in Indigenous children
Naylor et al. 2010 [30]	Canada	Explore the feasibility and acceptability of a school‐based program to improve inactivity and unhealthy eating patterns	Qualitative evaluation	Number of school kids who took part was not stated. 25 teachers and administrators in the three schools participated in delivering the intervention	School‐based: Action Schools! BC program is a model that provides tools for schools and teachers to create individualised action plans for PA and HE across six action zones: (i) school environment; (ii) scheduled PE; (iii) classroom action; (iv) family and community; (v) extra‐curricular; and (vi) school spirit	Follow‐up period not stated 76% retention rate	Thematic analysis	Outcome measures: feasibility and acceptability of the program Results: The program was feasible and well received, and all three schools implemented a mean of 140 min per week of physical activity and 2.3 times/week of healthy eating activities. No anthropometric measures were reported
Oosman 2012 (PhD Thesis) [31]	Canada	Evaluate the impact of a Métis culture‐based comprehensive school health program on PA, nutrition knowledge, attitudes, beliefs and behaviours of Métis children	Longitudinal study with an age‐matched controlled group	37 school children in all 8 and 9 years olds in classes 3 and 4 (16 in total in the treatment group)	School‐based: PA and Nutrition health promotion program. 17 culturally relevant PA and nutrition lessons were developed	4‐month timeframe 100% retention rate	Mix methods Wilcoxon test	Outcome measures: physical activity, nutrition, knowledge, attitudes and behaviours Results: Participating students spent significantly fewer minutes in sedentary activities than a comparison group post‐intervention, 495 min/day compared to 527 min/day, respectively. No statistically significant effect on nutrition. The health programming positively impacted student perceptions and awareness of healthy foods. No anthropometric measurements
Prapaveissis at al. 2022 [32]	New Zealand	Investigate empowerment and co‐design modules to build the capacity of Pasifika youth to develop community interventions for preventing prediabetes	Qualitative design (FGDs and IDIs)	Youth aged 15–24 from two communities (total participants = 41)	Community‐Based Participatory Research (CBPR) to develop Pasifika Prediabetes Youth Empowerment Programme' (PPYEP) has seven empowerment modules and five co‐design modules, (*n* = 12 modules) focused on increasing youth's leadership capacities and knowledge and skills about health	6‐months 71% retention rate	Thematic analysis	Outcome measures: knowledge and lifestyles Results: The programme increased youth's knowledge about healthy lifestyles, developed leadership and social change capacities and provided a tool to develop and refine culturally centred prediabetes‐prevention programmes
Ritenbaugh 2003 [33]	USA	Test the feasibility and efficacy of a high school‐based diabetes prevention intervention for at‐risk youth	Longitudinal study with three‐time points evaluation.	High school youth aged:16‐18 years Numbering 400 in total	School‐based diabetes prevention program. educational component targeting decreased consumption of sugared beverages, knowledge of diabetes risk factors and a youth‐oriented fitness centre to improve PA	3 years > 60% retention rate	Quantile regression	Outcome measures: Physical activity, sugary food/drink intake, plasma glucose, BMI, Insulin Results: Increase in PA, decrease in sugared soft drinks at school of about 4.8 oz/day/student. Plasma glucose levels did not change throughout the study. No difference in BMI between treatment and control. Both fasting and 30‐min plasma insulin levels significantly declined for the treatment group
Sauder et al. 2018 [34]	USA	Develop a culturally sensitive behavioural intervention (PA; games, cooking demonstrations and a group meal) for youth (Tribal Turning Point; TTP) (Feasibility study)	Randomised controlled trial	62 overweight/obese children: both sexes, ages 7–10 years	Community‐based: TTP program involving Active Learning group classes, youth/caregiver dyad MI sessions and a resource toolbox delivered by lay health coaches who were members of the tribes or non. Intervention arm (*n* = 29) attended 12 group classes (10–20 min) and five individual sessions, whereas the control arm (*n* = 33) attended three health and safety group sessions	8‐months 97% retention rate	Linear mixed models applying intention‐to‐treat analysis principles. Each model was adjusted for age, sex, tribe and baseline values (e.g., BMI analysis adjusted for BMI at baseline)	Outcome measures: feasibility, anthropometrics, insulin, glucose, HbA1c Results: The intervention benefitted anthropometric outcomes but not blood pressure and diabetes endpoints such as fasting insulin, glucose, HbA1c and HOMA‐IR
Seear et al. 2019 [35]	Australia	Adopting a locally designed diabetes prevention program and testing its acceptability, appropriateness, feasibility and sustainability	Qualitative	10 participants (18–38 years with 7 < 25 yrs).	Community‐based: locally adapted community‐led diabetes prevention program Sessions involved. An exercise circuit, outdoor cooking and education topics. healthy eating and physical activity and included graphic depictions of diabetes complications, including dialysis	6‐months	Qualitative thematic and content analysis	Outcome measures: feasibility, acceptability and appropriateness of program Results: The program was found to be acceptable and appropriate. Improvement in shopping choices portioning and reduction in soft drink consumption
Swanson et al. 2021 (PhD Thesis) [36]	USA	Determine the feasibility of a school‐based intervention designed to increase school‐aged children's knowledge regarding healthy lifestyles and T2DM prevention	Mainly qualitative	15 participants	School‐based: Diabetes Education in Tribal Schools (DETS) program incorporating lifestyle management options into the curriculum that specifically targets American Indian/Alaska Native (AI/AN) minority communities	No information of follow‐up period 100% retention rate	Qualitative analysis	Outcome measures: feasibility Results: the program was found to be feasible
Teufel et al. 1998 [37]	USA	Reduce T2DM risk factors in Zuni high school‐age youths	Multiple cross‐sectional studies for program evaluation	Intervention strategies target students, grades 9–12, attending the two high schools in Zuni: Zuni High School (ZHS), with an enrolment between 300 and 350 and Twin Buttes High School (TBHS), between 70 and 90	School‐based: Multicomponent intervention strategies were developed to target the risk factors: (1) supportive social networks, (2) wellness facility designed specifically for teens, (3) diabetes education integrated within the existing school curriculum and (4) modification of the food supply available to teens	4‐years No retention rate provided given the study design, is cross‐sectional and considers students in specific grades	Student *t*‐tests	Outcome measures: BMI, sugary drinks consumption, glucose/insulin Results: Reduction in BMI and sugary drinks consumption and an increase in glucose/insulin ratios suggest a decline in the incidence of hyperinsulinemia
Teufel‐shone et al. 2014 [38]	USA	Reduce T2DM risk factors among youths	Longitudinal study, with no randomisation	100 school youth (both sexes; mean age‐10.4 ± 1.1 in males and 10.4 ± 1.1 in females) in grades 3 through 8 were enrolled in the biweekly physical activity program, with each activity session lasting 45 to 60 min.	School‐based: Youth Wellness Program, led by three project personnel who were community members knowledgeable of local physical activity preferences	2‐years 71% retention rate	Paired sample *t*‐tests	Outcome measures: physical activity, BMI, blood glucose Results: Improvements in fitness measures improved fasting blood glucose levels but increased BMI

*Quality appraisal*: Two reviewers independently assessed study quality using a modified National Institute for Health (NIH) quality assessment tool for interventional and pre‐post studies [39]. Our adopted tool had five broad themes on which studies were appraised: study population and attrition (in the case of follow‐up studies), intervention implementation, outcome assessment, data analysis and confounding and reporting. The validity of our quality appraisal tool is equivalent to other available tools as particular attention was paid to all relevant factors, including reporting bias, selection bias, design features, statistical analysis and confounding bias [40]. Studies were scored a maximum of two points if they adequately satisfied a criterion; they were scored one if they had addressed a criterion partially and zero if there was no indication that they had addressed it. These points were then summed up and ranked. Our scoring aimed at rating studies relative to each other and did not necessarily capture all sources of bias. Therefore, studies with maximum scores do not imply that they are devoid of all sources of bias. However, as much as possible, an attempt was made to safeguard all critical sources of bias.

*Evidence synthesis*: Due to varied outcome measures and diversity of interventions, meta‐analysis was not possible. Instead, a narrative synthesis was employed to descriptively analyse interventions using the Joanna Briggs Institute's convergent integrated methodology for mixed methods systematic reviews [41]. This approach combines quantitative and qualitative data to provide a comprehensive understanding of the study topic. As a first step, data is extracted from all study types in a ‘qualitative’ approach through a narrative interpretation of findings in the text. Subsequently, all information was assembled, analysed and synthesised simultaneously and then integrated into a single set of findings, offering a deeper insight into the review topic [41].

## 4 Results

The search yielded a total of 1127 publications. Screening yielded 25 publications that met the inclusion criteria (Figure 1). Of these, 23 were peer‐reviewed journal articles, while the remaining two were theses or dissertations [31, 36].

These 25 publications were all from four countries, including the USA, Canada, New Zealand and Australia, with a total population of 4594 youth aged < 25 years. About 52% (*n* = 13) of the included studies were from the USA [14, 15, 16, 17, 18, 20, 21, 29, 33, 34, 36, 37, 38], 28% (*n* = 7) were from Canada [22, 23, 24, 25, 26, 30, 31] while the rest were from Australia (*n* = 3) [27, 28, 35] and New Zealand (*n* = 2) [19, 32]. Of note, most of the studies (80%) involved participants from North America (USA and Canada).

Most of the studies were quantitative studies (17 [65%]) [15, 16, 18, 20, 21, 22, 23, 25, 26, 27, 28, 29, 33, 34, 37, 38], while 5 (19%) were qualitative studies [17, 30, 32, 35, 36]. About four studies (16%) used mixed methods research involving quantitative and qualitative methods [14, 19, 24, 31]. Of the 25 studies, about 44% (*n* = 11) could be described as community‐based intervention studies [15, 17, 18, 20, 21, 23, 24, 29, 32, 34, 35]; another 10 (40%) were school‐based interventions [14, 19, 22, 27, 30, 31, 33, 36, 37, 38]. Three studies combined both school‐based and community‐based interventions [16, 25, 26]. One study was not specific, as it tested the effectiveness of a screening algorithm [28].

The study participants range from as small as 12 participants [24] to as high as 1500 participants [14]. More than half of the studies had a sample size of < 100 participants. The length of the study intervention ranged from 1.5 months [19] to as high as 48 months [37]. About 10 (40%) had interventions that were < 6 months, and 9 (36%) were between 6 months and 1 year. About 5 (20%) of the studies had interventions over 1 year [23, 26, 33, 37, 38]. Two studies did not report on the length of interventions [30, 36].

### 4.1 Outcome Measures

All included studies focused on interventions aimed at preventing or managing T2DM. A small number of studies additionally addressed interventions for other chronic conditions. Due to the diverse range of outcome measures used across studies to assess the effectiveness of the interventions, we categorise them into four main groups: (1) Knowledge and education‐based outcomes, (2) Behavioural outcomes (including dietary changes and physical activity), (3) Service‐related outcomes (including screening and detection programs), and (4) Anthropometric and biomarker outcomes. Table 2 describes the outcome categories.

**TABLE 2:** Description of outcomes used by studies in this review.

Category of outcome measures	Description
Knowledge‐based outcomes	Knowledge‐related outcomes included knowledge about T2DM, nutrition/healthy foods, attitudes and practices on dietary choices, diet quality, exercise and self‐efficacy and knowledge on healthy lifestyles [14, 15, 18, 22, 25, 27, 29, 31, 32, 33, 37]
Behavioural‐based and healthy dietary‐related outcomes	Outcomes around behaviour change mainly sort to increase Indigenous children and young adults' time on physical activity and reduce the length of time spent on sedentary activities and/or outcomes related to improving healthy dietary preferences, including low consumption of sugar‐sweetened beverages, red/processed meat, sodium and high consumption of fruit and vegetables [15, 19, 21, 23, 24, 26, 28, 30, 33, 34, 35, 36]
Health systems and services related outcomes	Some studies examined the effects of the interventions on improving testing and screening for early detection of T2DM among Indigenous children and young adults [23, 28]. Or Acceptability and appropriateness [18, 35] or feasibility [17, 24, 26, 30, 34, 35, 36] of program implementation
Anthropometric and biomarkers	Some studies assessed the effects of their interventions/programs on diabetes‐related anthropometric measures such as weight gain or loss, body mass index (BMI), BMI *z*‐scores, body fat percentage, fat‐free mass, waist circumference and blood biomarkers including fasting insulin, fasting blood glucose level, insulin resistance, VO2 max, HbA1c test, cholesterol levels, triglycerides (TG) level, blood pressure, glycemic control and glucose/insulin ratios [15, 16, 18, 19, 20, 22, 24, 25, 33, 34, 37, 38]

### 4.2 Quality of Studies

Tables S2 and S3 provide the quality appraisal tool and quality scores of all included studies, respectively. While none of the studies obtained all points on the quality appraisal tool, two studies scored the highest (20 points) [21, 22]. Studies were classified into three categories based on their relative scores: 0.0–0.4 for low quality, 0.5–0.7 for medium‐quality and 0.8–1.0 for high‐quality rating. This approach has been used by other systematic reviews [42] and is a helpful way of assessing relative quality among studies involved in a systematic review. About 8 (32%) of the 25 studies scored 0.8 or higher and were regarded as high‐quality studies [15, 16, 18, 21, 22, 23, 27, 34], another 12 (48%) studies were classified as medium quality [14, 19, 20, 24, 25, 26, 28, 31, 33, 35, 37, 38] and 5 (20%) studies were considered low‐quality [17, 29, 30, 32, 36].

All studies described their participants and recruited them from the same/similar population. Most studies did not specify inclusion and exclusion criteria, but a few provided sufficient information from which this could be inferred [14, 15, 16, 21, 22, 31, 37, 38]. Reviewed studies generally had low sample sizes. Indeed, more than half of the included studies reported < 100 study participants [15, 16, 17, 19, 20, 24, 27, 28, 29, 30, 31, 32, 34, 35, 36]. Only one study reported having over 1000 study participants [14]. Most studies reported a retention rate of 80% or above except for seven (7) studies that reported below 80% [20, 24, 28, 30, 32, 33, 38] and some five studies did not report on the retention rate at all [14, 26, 27, 35, 37].

Most studies described their interventions, including inputs, participants and duration except for two studies which did not provide information on the length of their intervention [30, 36]. Almost all studies did not provide sufficient information to ascertain if there was program contamination by other interventions. Except for two studies [14, 41], all reviewed studies described their outcome measures. Out of the 17 quantitative studies, only one did not describe statistical methods used to assess changes and based on the reviewers' assessments, did not use appropriate statistical methods [29]. Most studies did not evaluate the effect of confounding variables except for some four studies [21, 22, 23, 27]. Also, only seven studies considered the different gender of study participants in their analysis [22, 23, 26, 33, 34, 35, 37]. No systematic difference was observed between reported and unreported findings for almost all studies except for four studies that only reported areas of success [17, 29, 30, 36].

### 4.3 Effectiveness of Interventions

Eleven (44%) of the 25 reviewed studies reported positive improvements in knowledge‐based outcomes such as knowledge about diabetes and its risk factors, awareness of the importance of healthy nutrition (reducing the intake of sugary foods/drinks), physical activity among others [14, 15, 18, 22, 25, 27, 29, 31, 32, 33, 37]. Also, 12 (48%) studies reported improvements in behavioural‐related outcomes including improvements in healthy dietary habits, increases in physical activity/exercise and reduction in sedentary behaviour among others [15, 19, 21, 23, 25, 27, 31, 32, 33, 35, 37, 38], and 7 studies reported improvements in both knowledge‐based outcomes and behavioural‐related outcomes [15, 25, 27, 31, 32, 33, 37].

About 11 studies reported improvements related to service delivery‐related outcomes, including improvements in T2DM screening, cultural acceptability, feasibility and appropriateness of intervention for Indigenous youth [17, 18, 23, 24, 26, 28, 30, 33, 34, 35, 36]. Of the 12 studies that examined anthropometric and/or biomarker outcomes, three studies did not find any improvements in these measures [15, 24, 25]. This is not surprising as all three studies had an implementation period of < 5 months [15, 24, 25]. Four studies on the other hand found positive improvements in anthropometric/biomarkers such as weight, BMI and fasting blood glucose levels [18, 19, 20, 22], while the remaining four found improvements in some anthropometric/biomarker measures but not all measures assessed [16, 33, 34, 38].

Out of the 13 studies conducted in the USA, six reported improved knowledge‐based outcomes [14, 15, 18, 29, 33, 37] and five reported improvements in behaviour outcomes [15, 21, 33, 37, 38]. One study did not find any improvement in either knowledge or behavioural outcomes [34] and another one did not provide information on whether outcomes related to knowledge gain or behaviour were improved [17]. Two of the 13 studies from the USA indicated an improvement in anthropometric/biomarkers measures [18, 20]. At the same time, four other studies from the USA had a mixture of some positive improvements and no effects on some anthropometric/biomarkers [16, 33, 34, 38]. Among the studies from Canada, three found positive improvements in knowledge gained [22, 25, 31]. Three found improvements in behavioural outcomes [23, 25, 31], while four indicated improvements in service delivery‐related outcomes [23, 24, 26, 30]. Only one Canadian study reported positive improvements in anthropometric/biomarker measures [22], two did not find improvements in anthropometric/biomarkers measures [24, 25] and four did not include anthropometric/biomarkers as part of their outcome of interest [23, 26, 30, 31]. Among the three Australian studies included in this review, one reported improvement in both knowledge and behaviour outcomes [27], another reported improvement in service delivery [28] and the other reported improvements in both behaviour and service outcome measures [35]. Both studies from New Zealand found improvement in behaviour outcomes [19, 32], but one reported improvement in anthropometric/biomarkers measures [19]. While the other reported improvements in behaviour [32].

By study design, almost all quantitative studies reported positive improvements in either knowledge or behavioural‐related outcomes, with the exception of one quantitative study from the USA that did not find significant improvements in behaviour or knowledge but reported positive improvements in service delivery and anthropometric/biomarkers [34]. About three quantitative studies reported improvements in anthropometric/biomarker outcomes [18, 20, 22], and another four reported improvements in some of these outcomes [16, 33, 34, 38]. The remaining nine studies either did not find improvements or did not include anthropometric/biomarker outcomes in their evaluation.

Almost all qualitative studies reported improvements in either knowledge or behavioural‐related outcomes except one study that did not provide information on these outcomes [17]. Not surprisingly, none of the qualitative studies included anthropometric/biomarker measures in their evaluation. Three of the four mixed‐method studies indicated improvements in either behavioural or knowledge‐based outcomes [14, 19, 31]. The remaining study did not provide information to indicate whether these were improved or not [24]. One mixed method study reported an improvement in anthropometric/biomarker outcome [19], and another reported no improvement in anthropometric measurements [24], while two studies did not include these in their evaluation [14, 31].

### 4.4 Effectiveness Based on Intervention Characteristics

All school‐based interventions reported an improvement in either behavioural or knowledge‐based outcomes, while 9 out of 12 community‐based interventions reported improvements in either behavioural or knowledge‐based outcomes. Among the three studies that combined school‐based and community‐based interventions, one reported the feasibility of strategies for improving knowledge and behaviours associated with T2DM [26], another reported positive improvement in some anthropometric/biomarkers [16] and the other reported improvements in both knowledge‐based and behavioural outcomes [25]. Also, of the four school‐based interventions that reported on anthropometric/biomarker measures, all reported general enhancements [19, 22] or improvements in some anthropometric/biomarkers but not all anthropometric/biomarkers assessed [33, 38]. Also, four community‐based interventions reported effects on anthropometric/biomarker outcomes; three reported either general improvements in outcomes or improvements in some measures [18, 20, 34]. Two out of the five community‐based interventions that reported anthropometric/biomarker outcomes did not find any improvements in this regard [15, 24].

All interventions that were 1 year or more in duration were reported to be effective in improving either knowledge, behaviour or both. However, only two interventions < 6 months in duration that assessed changes in anthropometric/biomarker measures were found to be effective [19, 22]. For those between 6‐ and 12‐months duration who reported anthropometric/or biomarker measures, most reported improvements [16, 18, 20, 34]. In addition, two studies whose implementation duration was over a year reported positive improvements in anthropometric and/or biomarkers [33, 38]. Indicating the importance of intervention duration to achieving improvements in diabetes‐related risk factors.

Based on study quality, of the eight studies that were scored high quality, 7 (89%) were effective in improving outcomes related to either knowledge, behaviour or both and four reported positive improvements in anthropometric/biomarkers outcomes [16, 18, 22, 34]. However, none of the low‐quality studies reported anthropometric or biomarker outcomes and 4 out of 5 reported improved knowledge/behaviour‐related outcomes [29, 30, 32, 36].

Based on the study setting, 20 studies were primarily implemented in rural settings; of this number, five reported improvements in both knowledge‐based outcomes and behaviour‐related outcomes [15, 25, 31, 33, 37], four reported improvements in knowledge‐based outcomes [14, 18, 22, 29], another four reported improvements in behaviour related outcomes alone [21, 23, 35, 38], seven studies reported improvements in anthropometric or biomarker outcomes [18, 20, 22, 33, 34, 37, 38] and 10 improved health service‐related outcomes within rural settings [17, 18, 23, 26, 28, 30, 33, 34, 35, 36]. Of the four interventions mainly implemented in urban settings, one reported improvement in knowledge‐based outcomes [27], two reported improvements in behaviour‐related outcomes [19, 27] and two reported improvements in anthropometric or biomarker outcomes [16, 19], while one improved health service‐related outcomes within these urban settings [24]. One study that was implemented in both rural and urban settings showed positive outcomes for knowledge and behaviour but none for anthropometric measures [32].

## 5 Discussion

This review examines the current available evidence on interventions for preventing or managing T2DM among Indigenous youth. The review identified 25 intervention studies using a wide range of strategies and implemented either as community/home‐based interventions, school‐based interventions or a combination of both. Studies used various methods in implementing and evaluating their programs that involved quantitative, qualitative and mixed methods approaches involving both quantitative and qualitative methods. Notably, most of the identified studies were from North America (USA and Canada), with very few studies from Australia and New Zealand.

It was found that most studies focused on the effect of their intervention on improving knowledge, behaviour and service delivery aimed at reducing T2DM risk factors among Indigenous youth. However, less than half of the programs specifically targeted intervention effects on proximal risk factors for T2DM, including anthropometrics, adiposity and diabetes biomarkers such as fasting insulin and blood glucose levels, about program implementation to address diabetes, with less consistent results [15, 16, 18, 19, 20, 22, 24, 25, 33, 34, 38].

All school‐based interventions reported improvements in either behavioural or knowledge‐based outcomes. Schools offer unique opportunities to promote health and well‐being among youth through diverse curricular and extracurricular activities. Effective school‐based interventions for preventing and managing T2DM in Indigenous youth were those that adapted school curricula and programs tailored to address the specific needs of this group of young people. These adaptations consider the cultural and social contexts of Indigenous youth and their communities [14]. A key characteristic of effective school‐based interventions is the use of curriculums that integrate science and traditional cultural learning practices such as storytelling, games, food testing, experiments, puppet shows, crafts and audiovisual to educate students about science, diabetes and its risk factors and the importance of nutrition and physical activity in maintaining health and balance in life [14, 25, 27]. School‐based interventions that apply an inquiry‐based approach to learning, building skills in observation, measurement, prediction, experimentation and communication and providing healthy lifestyle messages, using traditional foods and those commonly eaten in the community while being flexible and adaptive were key strategies for successful development and implementation of school‐based interventions for T2DM prevention and management [26, 30].

Successful community‐based interventions mostly use community‐based participatory research to actively engage community members and build trusting partnerships. This collaborative approach ensured that the interventions addressed the specific needs and priorities of Indigenous youth [15]. Key strategies for success involved the incorporation of cultural and traditional activities such as berry picking, horseback riding, dancing, hunting, hiking and camping. The use of storytelling and native language to convey information enhanced cultural relevance and understanding [15, 29, 34]. Engaging youth in practical hands‐on activities such as preparing and testing healthy snacks, learning and participating in traditional native games such as double ball, ring the stick, run and scream and shiny keeping and discussing weekly activity and nutrition diaries made learning enjoyable and relevant [15, 35].

The review found that interventions that combined both school‐based and community‐based strategies were more likely to be successful in improving knowledge, behaviour and service delivery aimed at reducing T2DM risk factors among Indigenous children and youth [16, 25].

Our review highlights the crucial role of involving Indigenous community leaders in the development and implementation of these interventions [17, 26, 34, 35]. Community leaders are essential stakeholders. They ensure culturally appropriate interventions are implemented through this participation. A collaborative approach involves Indigenous people from various backgrounds (e.g., health professionals, community leaders and tribal leaders). By placing Indigenous people in leading roles during program implementation, interventions can be effectively delivered for the prevention and management of T2DM in Indigenous youth [17, 26, 34, 35].

It is worthy of note that the effectiveness of these interventions also requires addressing underlying social determinants of health, such as poverty, low educational attainment, unemployment and socioeconomic marginalisation, which limit access to healthcare, healthy eating choices and physical activities and contribute to the burden of diabetes and other chronic diseases among Indigenous people [6, 10].

## 6 Study Limitations

This study has some limitations; first, although an extensive literature search was done, a few interventions may have been missed within the grey literature. Second, as few studies combined school‐based and community‐based strategies, the evidence on the effectiveness of a combined approach to either of them separately is not conclusive. Furthermore, most of the included studies involved a very small sample size and were implemented over short time periods making their effectiveness unclear. Also, due to limitations in the range of outcome variables reported by included studies, we could not gauge the effects of study interventions on some outcome variables such as quality of life and mental health disorders.

## 7 Conclusion

The prevention and management of T2DM among children and young Indigenous peoples require culturally appropriate interventions designed to target both knowledge, lifestyle as well as dietary choices. A life course approach to the prevention and management of diabetes measures from a young age is required to reduce the risk of diabetes and other chronic cardiometabolic conditions among Indigenous people. Interventions need to be implemented within and beyond the health system (including educational settings, places of employment and homes) through government and non‐government agencies and need to commence as early as possible in the life course.

Stronger scientific evidence is warranted in young Indigenous people to inform public health policy and clinical practice in efforts to prevent and manage chronic diseases in this segment of Indigenous people. There is a need for more of such interventions in Australia and New Zealand to help generate country‐based evidence for improving the health and well‐being of Indigenous youth.

## Author Contributions

Conception and design: A.A.M., E.W.K. and T.W.U. database search and screening: E.W.K. and T.W.U. data extraction, analysis and interpretation: E.W.K., Y.F., T.W.U. and A.A.M. writing – original draft: E.W.K. and A.A.M. writing – review: Y.F., T.W.U., T.L.D., R.V.S., A.A.M. and E.W.K.

## Conflicts of Interest

The authors declare no conflicts of interest.

## Supporting information


Data S1.

## Data Availability

All data used in this paper are contained within the paper and accompanying Supporting Information.

## References

[1] World Bank , “Indigenous Peoples Overview,” (2022), https://www.worldbank.org/en/topic/indigenouspeoples.

[2] World Health Organisation , “Indigenous Peoples and Tackling Health Inequities: WHO Side Event at the 21st Session of the UN Permanent Forum on Indigenous Issues,” (2022), https://www.who.int/news‐room/events/detail/2022/05/03/default‐calendar/indigenous‐peoples‐and‐tackling‐health‐inequities‐‐who‐side‐event‐at‐the‐2022‐session‐of‐the‐un‐permanent‐forum‐on‐indigenous‐issues.

[3] K. L. Ong , L. K. Stafford , S. A. McLaughlin , et al., “Global, Regional, and National Burden of Diabetes From 1990 to 2021, With Projections of Prevalence to 2050: A Systematic Analysis for the Global Burden of Disease Study 2021,” Lancet 402 (2023): 203–234. 37356446 10.1016/S0140-6736(23)01301-6 PMC10364581

[4] J. E. Lucero and Y. Roubideaux , “Advancing Diabetes Prevention and Control in American Indians and Alaska Natives,” Annual Review of Public Health 43 (2022): 461–475. 10.1146/annurev-publhealth-093019-010011 PMC9924140 35380066

[5] H. D. Nguyen , S. Chitturi , and L. J. Maple‐Brown , “Management of Diabetes in Indigenous Communities: Lessons From the Australian Aboriginal Population,” Internal Medicine Journal 46 (2016): 1252–1259. 27130346 10.1111/imj.13123

[6] T. C. Turin , N. Saad , M. Jun , et al., “Lifetime Risk of Diabetes Among First Nations and Non‐First Nations People,” CMAJ 188 (2016): 1147–1153. 27647609 10.1503/cmaj.150787 PMC5088075

[7] D. Simmons , J. Voyle , E. Rush , and M. Dear , “The New Zealand Experience in Peer Support Interventions Among People With Diabetes,” Family Practice 27 (2009): I53–I61. 19254967 10.1093/fampra/cmp012

[8] E. A. Sellers , K. Moore , and H. J. Dean , “Clinical Management of Type 2 Diabetes in Indigenous Youth,” Pediatric Clinics of North America 56 (2009): 1441–1459. 19962030 10.1016/j.pcl.2009.09.013

[9] P. Azzopardi , A. D. Brown , P. Zimmet , et al., “Type 2 Diabetes in Young Indigenous Australians in Rural and Remote Areas: Diagnosis, Screening, Management and Prevention,” Medical Journal of Australia 197 (2012): 32–36. 22762229 10.5694/mja12.10036

[10] F. B. Hu , M. J. Stampfer , S. M. Haffner , C. G. Solomon , W. C. Willett , and J. A. E. Manson , “Elevated Risk of Cardiovascular Disease Prior to Clinical Diagnosis of Type 2 Diabetes,” Diabetes Care 25 (2002): 1129–1134. 12087009 10.2337/diacare.25.7.1129

[11] B. T. Johnson and E. A. Hennessy , “Systematic Reviews and Meta‐Analyses in the Health Sciences: Best Practice Methods for Research Syntheses,” Social Science and Medicine 233 (2019): 237–251. 31233957 10.1016/j.socscimed.2019.05.035 PMC8594904

[12] D. Moher , A. Liberati , J. Tetzlaff , and D. G. Altman , “Preferred Reporting Items for Systematic Reviews and Meta‐Analyses: The PRISMA Statement,” PLoS Medicine 6 (2009): e1000097. 19621072 10.1371/journal.pmed.1000097 PMC2707599

[13] J. Bullen , T. Hill‐Wall , K. Anderson , et al., “From Deficit to Strength‐Based Aboriginal Health Research—Moving Toward Flourishing,” International Journal of Environmental Research and Public Health 20 (2023): 5395. 37048008 10.3390/ijerph20075395 PMC10094537

[14] L. Aho , J. Ackerman , S. Bointy , et al., “Health Is Life in Balance: Students and Communities Explore Healthy Lifestyles in a Culturally Based Curriculum,” Pimatisiwin 8 (2011): 151–168. 22279450 PMC3263817

[15] B. Brown , C. Noonan , K. J. Harris , et al., “Developing and Piloting the Journey to Native Youth Health Program in Northern Plains Indian Communities,” Diabetes Educator 39 (2013): 109–118. 23150531 10.1177/0145721712465343

[16] A. Carrel , A. Meinen , C. Garry , and R. Storandt , “Effects of Nutrition Education and Exercise in Obese Children: The Ho‐Chunk Youth Fitness Program,” WMJ 104 (2005): 44–47. 16138515

[17] J. Q. Chadwick , M. A. Tullier , L. Wolbert , et al., “Collaborative Implementation of a Community‐Based Exercise Intervention With a Partnering Rural American Indian Community,” Clinical Trials 16 (2019): 391–398. 30939923 10.1177/1740774519839066 PMC6663565

[18] R. Chambers , S. Rosenstock , M. Walls , et al., “Engaging Native American Caregivers in Youth‐Focused Diabetes Prevention and Management,” Preventing Chronic Disease 15 (2019): E85. 10.5888/pcd15.170521 PMC6016401 29935076

[19] Y. Chansavang , C. R. Elley , B. McCaffrey , C. Davidson , O. Dewes , and L. Dalleck , “Feasibility of an After‐School Group‐Based Exercise and Lifestyle Programme to Improve Cardiorespiratory Fitness and Health in Less‐Active Pacific and Maori Adolescents,” Journal of Primary Health Care 7 (2015): 57–64. 25770717

[20] V. Shah , L. Colip , M. R. Burge , et al., “Exercise Intervention Improves the Metabolic Profile and Body Composition of South Western American Indian Adolescents,” Journal of Diabetes and Obesity 3 (2016): 1–7. 10.15436/2376-0494.16.1180 PMC5400367 28435884

[21] K. Ducharme‐Smith , R. Chambers , V. Garcia‐Larsen , et al., “Native Youth Participating in the Together on Diabetes 12‐Month Home‐Visiting Program Reported Improvements in Alternative Healthy Eating Index‐2010 Diet Quality Domains Likely to be Associated With Blood Pressure and Glycemic Control,” Journal of the Academy of Nutrition and Dietetics 121 (2021): 1125–1135. 33547030 10.1016/j.jand.2020.12.017

[22] P. Eskicioglu , J. Halas , M. Seńéchal , et al., “Peer Mentoring for Type 2 Diabetes Prevention in First Nations Children,” Pediatrics 133 (2014): e1624–e1631. 24819579 10.1542/peds.2013-2621

[23] K. L. Frejuk , O. Harasemiw , P. Komenda , et al., “Impact of a Screen, Triage and Treat Program for Identifying Chronic Disease Risk in Indigenous Children,” CMAJ 193 (2021): E1415–E1422. 34518342 10.1503/cmaj.210507 PMC8443280

[24] E. Huynh , D. Rand , C. McNeill , et al., “Beating Diabetes Together: A Mixed‐Methods Analysis of a Feasibility Study of Intensive Lifestyle Intervention for Youth With Type 2 Diabetes,” Canadian Journal of Diabetes 39 (2015): 484–490. 26553586 10.1016/j.jcjd.2015.09.093

[25] K. E. Kakekagumick , M. N. Hayward , S. B. Harris , et al., “Sandy Lake Health and Diabetes Project: A Community‐Based Intervention Targeting Type 2 Diabetes and Its Risk Factors in a First Nations Community,” Frontiers in Endocrinology 4 (2013): 170. 24302919 10.3389/fendo.2013.00170 PMC3824247

[26] A. C. Macaulay , G. Paradis , L. Potvin , et al., “The Kahnawake Schools Diabetes Prevention Project: Intervention, Evaluation, and Baseline Results of a Diabetes Primary Prevention Program With a Native Community in Canada,” Preventive Medicine 26 (1997): 779–790. 9388789 10.1006/pmed.1997.0241

[27] C. Malseed , A. Nelson , R. Ware , C. Malseed , A. Nelson , and R. Ware , “Evaluation of a School‐Based Health Education Program for Urban Indigenous Young People in Australia,” Health 6 (2014): 587–597.

[28] A. Manifold , D. Atkinson , J. V. Marley , et al., “Complex Diabetes Screening Guidelines for High‐Risk Adolescent Aboriginal Australians: A Barrier to Implementation in Primary Health Care,” Australian Journal of Primary Health 25 (2019): 501–508. 31634436 10.1071/PY19030

[29] E. Marlow , G. D'eramo Melkus , and A. M. Bosma , “STOP Diabetes! An Educational Model for Native American Adolescents in the Prevention of Diabetes,” Diabetes Educator 24 (1998): 441–450. 9830948 10.1177/014572179802400403

[30] P. J. Naylor , J. Scott , J. Drummond , L. Bridgewater , H. A. McKay , and C. Panagiotopoulos , “Implementing a Whole School Physical Activity and Healthy Eating Model in Rural and Remote First Nations Schools: A Process Evaluation of Action Schools! BC,” Rural and Remote Health 10 (2010): 1296. 20476839

[31] S. Oosman , “Kica‐Wasimisinanahk Miyo‐Ayawin—Our Children's Health. Promoting Physical Activity and Nutrition Through a Health Promoting School‐Based Intervention in a Métis Community,” (2012).

[32] D. Prapaveissis , A. Henry , E. Okiama , et al., “Assessing Youth Empowerment and Co‐Design to Advance Pasifika Health: A Qualitative Research Study in New Zealand,” Australian and New Zealand Journal of Public Health 46 (2022): 56–61. 34821440 10.1111/1753-6405.13187 PMC9936602

[33] C. Ritenbaugh , N. I. Teufel‐Shone , M. G. Aickin , et al., “A Lifestyle Intervention Improves Plasma Insulin Levels Among Native American High School Youth,” Preventive Medicine 36 (2003): 309–319. 12634022 10.1016/s0091-7435(02)00015-4

[34] K. A. Sauder , D. Dabelea , R. Bailey‐Callahan , et al., “Targeting Risk Factors for Type 2 Diabetes in American Indian Youth: The Tribal Turning Point Pilot Study,” Pediatric Obesity 13 (2018): 321–329. 28635082 10.1111/ijpo.12223 PMC5740022

[35] K. H. Seear , D. N. Atkinson , M. P. Lelievre , L. M. Henderson‐Yates , and J. V. Marley , “Piloting a Culturally Appropriate, Localised Diabetes Prevention Program for Young Aboriginal People in a Remote Town,” Australian Journal of Primary Health 25 (2019): 495–500. 31581978 10.1071/PY19024

[36] L. M. Swanson , “Diabetes Education Among American Indians on the Fort Berthold Indian Reservation: Improving Educational Interventions in the School Setting,” (2021).

[37] N. I. Teufel and C. K. Ritenbaugh , “Development of a Primary Prevention Program: Insight Gained in the Zuni Diabetes Prevention Program,” Clinical Pediatrics 37 (1998): 131–142, 10.1177/000992289803700211. 9492122

[38] N. I. Teufel‐Shone , M. Gamber , H. Watahomigie , T. J. Siyuja , L. Crozier , and S. L. Irwin , “Using a Participatory Research Approach in a School‐Based Physical Activity Intervention to Prevent Diabetes in the Hualapai Indian Community, Arizona, 2002–2006,” Preventing Chronic Disease 11 (2014): E166. 25254984 10.5888/pcd11.130397 PMC4176473

[39] NIH , “Study Quality Assessment Tools,” (2020), https://www.nhlbi.nih.gov/health‐topics/study‐quality‐assessment‐tools.

[40] S. Sanderson , I. D. Tatt , and J. P. T. Higgins , “Tools for Assessing Quality and Susceptibility to Bias in Observational Studies in Epidemiology: A Systematic Review and Annotated Bibliography,” International Journal of Epidemiology 36 (2007): 666–676. 17470488 10.1093/ije/dym018

[41] C. Stern , L. Lizarondo , J. Carrier , et al., “Methodological Guidance for the Conduct of Mixed Methods Systematic Reviews,” JBI Evidence Implementation 19 (2021): 120–129. 34061049 10.1097/XEB.0000000000000282

[42] E. W. Kanmiki , Y. Fatima , and A. A. Mamun , “Multigenerational Transmission of Obesity: A Systematic Review and Meta‐Analysis,” Obesity Reviews 23 (2022): e13405. 34970828 10.1111/obr.13405

