# Supplementary material for: Interventions for Type 2 Diabetes Prevention and Management Among Indigenous Children and Youth: A Systematic Review
Source: Endocrinol Diabetes Metab. 2025 Jan 13;8(1):e70026. doi: 10.1002/edm2.70026 (PMC11726261; doi:10.1002/edm2.70026)
Supplement: Supplementary file 1 — Data S1. [file EDM2-8-e70026-s001.docx]

**Supplementary Materials**

S1: Search Terms

| **Proposed Database to Search** | Pubmed, Embase, Web of Science (core collection), ProQuest, Scopus, PsycINFO |
| --- | --- |
| **PICO** | **Search Terms** |
|  |  |
| **Population** | indigenous OR north american indian OR metis OR inut* OR torres strait islander OR first nation OR maori OR aborigin* OR native hawaiian OR oceanic ancestry group OR australoid race OR pacific island america OR native OR north american amerind OR eskimo OR alaska native* OR aleut OR inupiat OR kalaallit OR first nation OR kanaka maoli OR yupik |
| **Sub population** | youth OR young adult* OR adolescen* OR children OR childhood OR child OR young people OR young OR kids OR less than 25years |
| **Condition** | Diabetes OR diabetes mellitus OR high blood sugar OR sugar diabetes OR high blood glucose OR type 2 diabetes OR TYPE 2 DIABETES OR T2D OR type II diabetes mellitus OR DMII OR type II diabetes OR non-insulin dependent diabetes mellitus OR NIDDM insulin resistance OR fasting plasma glucose OR prediabetes OR impaired glucose tolerance OR type II diabetes |
| **Intervention/ Strategies** | Prevention OR prevent OR preventive OR avoidance OR avoid OR management OR screen OR screening OR treatment OR program* OR intervention OR control OR strategies OR care protocols OR therapy OR therapeutics |
| **Context (Countries)** | **Australia OR New Zealand OR Canada OR United State of America OR USA** |

**Table S2: Quality Assessment tool for the study on interventions for preventing and managing type 2 diabetes among Indigenous children and young adults (Adopted from NIH Quality Assessment tool for Controlled Interventions and before-after studies )**

| **Criteria** | **Yes** | | **No**  (0) | **N/A** |
| --- | --- | --- | --- | --- |
|  | **Completely**  **(2)** | **Partially**  **(1)** |  |  |
| **Study Population & Attrition:** | | | | |
| 1. Was the study population clearly specified & defined? |  |  |  |  |
| 2. Were all the subjects selected or recruited from the same or similar populations (including the same time period)? |  |  |  |  |
| 3. Were inclusion and exclusion criteria for being in the study prespecified and applied uniformly to all participants? |  |  |  |  |
| 4. Were the participants in the study representative of those who would be eligible for the intervention in the population of interest? |  |  |  |  |
| 5. Was the overall drop-out rate from the study at endpoint 20% or lower of the number allocated to treatment? |  |  |  |  |
| 6. Is there absence of a systematic difference in loss of participants/ participant data or drop out or missing data |  |  |  |  |
| **Implementation of Intervention** | | | | |
| 1. Was the intervention clearly described (main inputs, personnel involved, length/duration) |  |  |  |  |
| 2. Was there high adherence to the intervention protocols for each treatment group? |  |  |  |  |
| 3. Were other interventions avoided or similar in the groups (e.g., similar background treatments)? |  |  |  |  |
| 4. Did the authors report that the sample size was sufficiently large to be able to detect a difference in the main outcome between groups with at least 80% power? |  |  |  |  |
| 6. Were outcomes reported or subgroups analyzed prespecified (i.e., identified before analyses were conducted)? |  |  |  |  |
| **Outcome Assessment** | | | | |
| 1. Were the outcome measures prespecified, clearly defined, valid, reliable, and assessed consistently across all study participants? |  |  |  |  |
| 2. Did the statistical methods examine changes in outcome measures from before to after the intervention? Were statistical tests done that provided p values for the pre-to-post changes? |  |  |  |  |
| 3. Were outcome measures of interest taken multiple times before the intervention and multiple times after the intervention (i.e., did they use an interrupted time-series design)? |  |  |  |  |
| **Data Analysis & Confounding** | | | | |
| 1. Were appropriate statistical methods use in analysing the data? |  |  |  |  |
| 2. Were key potential confounding variables measured and adjusted statistically for their impact on the outcome measures? |  |  |  |  |
| 3. Does the analysis take into consideration different gender of study participants |  |  |  |  |
| **Reporting** |  |  |  |  |
| 1. Is there any systematic difference between reported and unreported findings in teams of outcomes /or incomplete reporting of findings? |  |  |  |  |

*This tool is an adopted version of NIH Quality Assessment Tool for interventions studies and pre-post Studies. Scoring would be done from a maximum of two (2) to zero (0).* *Studies would be scored 2 if they have adequately satisfied a criteria, 1 if they have addressed a criteria partially and zero if they have not addressed the criteria or if they is no indication is been addressed*

**Table S3: Quality Assessment Scores**

| **Study** | **Study Population & Attrition:** | | | | | | **Implementation of Intervention** | | | | | **Outcome Assessment** | | | **Data Analysis & Confounding** | | | **Reporting** | **SCORE** | | |
| --- | --- | --- | --- | --- | --- | --- | --- | --- | --- | --- | --- | --- | --- | --- | --- | --- | --- | --- | --- | --- | --- |
|  |  |  |  |  |  |  |  |  |  |  |  |  |  |  |  |  |  |  | **Sum score** | | |
| **Aho et al 2011** | 2 | 1 | 1 | 1 | 1 | 1 | 1 | 1 | 1 | 0 | 0 | 0 | 1 | 0 | 1 | 0 | 0 | 1 | 13 | 0.7 |  |
| **Brown et al 2013** | 2 | 1 | 1 | 1 | 1 | 1 | 2 | 1 | 1 | 0 | 0 | 1 | 1 | 0 | 1 | 0 | 0 | 1 | 15 | 0.8 |  |
| **Carrel et al 2005** | 2 | 1 | 1 | 1 | 2 | 2 | 2 | 2 | 1 | 0 | 1 | 1 | 1 | 0 | 1 | 0 | 0 | 1 | 19 | 1.0 |  |
| **Chadwick et al 2019** | 1 | 1 | 0 | 1 | 1 | 1 | 1 | 1 | 0 | 0 | 1 | 0 | 0 | 0 | 0 | 0 | 0 | 0 | 8 | 0.4 |  |
| **Chambers et al 2018** | 1 | 1 | 0 | 1 | 2 | 1 | 1 | 1 | 1 | 0 | 1 | 1 | 1 | 1 | 1 | 0 | 0 | 1 | 15 | 0.8 |  |
| **Chansavang et al 2015** | 1 | 1 | 0 | 0 | 1 | 1 | 1 | 1 | 1 | 0 | 1 | 1 | 1 | 1 | 1 | 0 | 0 | 1 | 13 | 0.7 |  |
| **Colip et al 2016** | 1 | 1 | 0 | 0 | 1 | 1 | 1 | 1 | 1 | 0 | 0 | 1 | 1 | 0 | 1 | 0 | 0 | 1 | 11 | 0.6 |  |
| **Costa-Urrutia et al 2019** | 1 | 1 | 1 | 0 | 1 | 2 | 1 | 1 | 0 | 0 | 1 | 1 | 1 | 0 | 2 | 2 | 2 | 2 | 19 | 1.0 |  |
| **Ducharme-Smith et al 2021** | 1 | 1 | 1 | 1 | 1 | 1 | 2 | 1 | 0 | 1 | 1 | 1 | 2 | 1 | 2 | 2 | 0 | 1 | 20 | 1.0 |  |
| **Eskicioglu et al 2014** | 1 | 1 | 1 | 1 | 1 | 1 | 1 | 1 | 1 | 1 | 1 | 2 | 1 | 0 | 2 | 2 | 1 | 1 | 20 | 1.0 |  |
| **Frejuk et al 2021** | 1 | 1 | 0 | 1 | 1 | 1 | 1 | 1 | 1 | 0 | 1 | 1 | 1 | 0 | 1 | 1 | 1 | 1 | 15 | 0.8 |  |
| **Huynh et al 2015** | 1 | 1 | 0 | 0 | 1 | 0 | 1 | 1 | 0 | 0 | 0 | 1 | 1 | 0 | 1 | 0 | 0 | 1 | 9 | 0.5 |  |
| **Kakekagumick et al 2013** | 1 | 1 | 0 | 1 | 1 | 1 | 1 | 0 | 0 | 0 | 0 | 1 | 1 | 0 | 1 | 0 | 0 | 1 | 10 | 0.5 |  |
| **Macaulay et al 1997** | 1 | 1 | 0 | 1 | 1 | 1 | 1 | 1 | 0 | 0 | 0 | 2 | 1 | 0 | 1 | 0 | 1 | 1 | 13 | 0.7 |  |
| **Malseed et al 2014** | 1 | 1 | 0 | 1 | 1 | 1 | 1 | 1 | 0 | 0 | 0 | 1 | 2 | 1 | 2 | 2 | 0 | 1 | 16 | 0.8 |  |
| **Manifold et al 2019** | 1 | 1 | 0 | 0 | 0 | 0 | 1 | 1 | 0 | 0 | 0 | 1 | 1 | 1 | 1 | 0 | 0 | 1 | 9 | 0.5 |  |
| **Marlow et al 1998** | 1 | 1 | 0 | 0 | 1 | 1 | 1 | 1 | 0 | 0 | 1 | 1 | 0 | 0 | 0 | 0 | 0 | 0 | 8 | 0.4 |  |
| **Naylor et al 2010** | 1 | 1 | 0 | 0 | 0 | 1 | 1 | 1 | 0 | 0 | 0 | 1 | 0 | 0 | 0 | 0 | 0 | 0 | 6 | 0.3 |  |
| **Oosman 2012** | 1 | 1 | 1 | 1 | 1 | 1 | 1 | 1 | 0 | 0 | 0 | 1 | 0 | 0 | 1 | 0 | 0 | 1 | 11 | 0.6 |  |
| **Prapaveissis at al 2022** | 1 | 1 | 0 | 0 | 0 | 0 | 1 | 1 | 0 | 0 | 0 | 1 | 0 | 0 | 0 | 0 | 0 | 1 | 6 | 0.3 |  |
| **Ritenbaugh 2003** | 1 | 1 | 0 | 1 | 0 | 1 | 1 | 1 | 0 | 1 | 1 | 1 | 1 | 1 | 1 | 0 | 1 | 1 | 14 | 0.7 |  |
| **Sauder et al 2018** | 1 | 1 | 0 | 1 | 1 | 1 | 1 | 1 | 1 | 1 | 1 | 1 | 1 | 0 | 1 | 0 | 1 | 1 | 15 | 0.8 |  |
| **Seear et al 2019** | 1 | 1 | 0 | 0 | 1 | 1 | 1 | 1 |  | 0 | 1 | 1 | 0 | 0 | 0 | 0 | 1 | 1 | 10 | 0.5 |  |
| **Swanson et al 2021** | 1 | 1 | 0 | 0 | 1 | 1 | 1 | 1 | 0 | 0 | 0 | 1 | 0 | 0 | 0 | 0 | 0 | 0 | 7 | 0.4 |  |
| **Teufel et al 1998** | 1 | 1 | 1 | 0 | 1 | 1 | 1 | 0 | 1 | 0 | 1 | 1 | 1 | 0 | 1 | 0 | 1 | 1 | 13 | 0.7 |  |
| **Teufel-shone et al 2014** | 1 | 1 | 1 | 1 | 0 | 0 | 1 | 1 | 1 | 0 | 0 | 1 | 1 | 0 | 1 | 0 | 0 | 1 | 11 | 0.6 |  |
